# Contrast-Enhanced Ultrasound Evaluation of Mifepristone for Treatment of Low-Risk Cesarean Scar Pregnancy

**DOI:** 10.1155/2020/3725353

**Published:** 2020-10-31

**Authors:** Xi Xiong, Chun-yan Gao, De-mei Ying, Ping Yan, Zhi-jia Zhang, Na Kuang, Hong-ju Tian, Li Luo, Shu-yu Long, Zheng-qiong Chen

**Affiliations:** ^1^Department of Obstetrics and Gynecology, Second Clinical Medical College of Army Medical University, Chongqing 400037, China; ^2^Department of Clinical Laboratory, Second Clinical Medical College of Army Medical University, Chongqing 400037, China

## Abstract

**Purpose:**

The effect of mifepristone for treatment of low-risk cesarean scar pregnancy (CSP) was monitored by contrast-enhanced ultrasound (CEUS).

**Methods:**

Data were collected from 23 CSP patients with a 10-point risk score <5 (low-risk CSP) and from 23 intrauterine pregnancy (IUP) patients with a scar from a previous cesarean delivery. All patients were prescribed 75 mg mifepristone daily for 2 days and underwent transvaginal CEUS before and after administration of mifepristone. On the third day, uterine curettage was performed after transvaginal CEUS. Arrival time (AT), peak intensity (PI), and area under the curve (AUC) around the gestational sac were monitored by CEUS before and after application of mifepristone, and the rate of effective treatment was compared between the two patient groups.

**Results:**

No patients experienced side effects from either the CEUS procedure or the mifepristone treatment. Changes in AT, PI, and AUC index from before vs. after mifepristone treatment did not differ significantly between the two groups (all *p* values >0.05). There was also no significant difference in the rate of effective treatment between the two groups (95.65% in the CSP group vs. 100% in the IUP group; *p* > 0.05).

**Conclusions:**

Based on monitoring by CEUS, the effect of mifepristone in low-risk CSP was comparable to that in IUP.

## 1. Introduction

Cesarean scar pregnancy (CSP), or implantation of the gestational sac in a hysterotomy scar, is a rare but serious complication that can occur in a subsequent pregnancy after cesarean delivery [1]; it is especially concerning in China [2]. Prenatal diagnosis of CSP is based on the presence of a gestational sac at the site of the previous uterine incision and the presence of an empty uterine cavity and cervix and thin myometrium adjacent to the bladder [3]. The severity of CSP has been found to correlate with clinical and sonographic characteristics including the implantation site, blood flow around the gestational sac, timing within gestation, and number of previous cesarean deliveries [4, 5]. Numerous management options for CSP have been evaluated based on case series, including laparoscopy, uterine artery embolization (UAE), and high-intensity focused ultrasound (HIFU) [6–8]. However, no standardized diagnostic or management guidelines have been published [9].

Our group has developed and validated a scoring system to rate the severity of CSP on a 10-point scale based on clinical indicators including thickness of the myometrium at uterine incision, grading of blood flow, fetal heartbeat, location of the gestational sac, maximal diameter of the gestational sac, and number of previous cesarean sections [10]. To validate this scoring system, patients were assigned a risk score based on these indicators, and treatment modalities employed were then assessed in relation to risk scores. Results showed that patients with CSP risk scores lower than 5 were significantly less likely to need invasive salvage treatments compared to higher-risk patients [10].

Mifepristone is the most commonly used progesterone antagonist [11]. In addition to producing prostaglandins to accelerate the degeneration and necrosis of villi, mifepristone can also reduce the vascular endothelial growth factor in decidual tissue, thereby reducing blood supply to the embryo and bringing about termination of the pregnancy [12]. A case report showed that the approach with mifepristone for treatment of CSP may be a safer and less invasive method [13]. By contrast, another study suggested that mifepristone is not very effective in the treatment of CSP [14]. Therefore, there are no commonly accepted clinical management guidelines on the use of mifepristone for CSP.

Contrast-enhanced ultrasound (CEUS) is a safe, widely available, and relatively inexpensive imaging technique that uses dedicated imaging ultrasound sequences and FDA-approved contrast microbubbles, permitting high diagnostic accuracy [15]. CEUS is a convenient method for diagnosis of CSP, has excellent spatial and temporal resolution, and can be used for quantitative assessment of microcirculation perfusion of the gestational sac [16]. Building on findings from our previous work, we sought to quantitatively analyze changes in microcirculation around the gestational sac using CEUS in order to evaluate the efficacy of mifepristone in the treatment of low-risk CSP.

## 2. Materials and Methods

Data were prospectively collected between July 2018 and March 2019 from patients seen in the department of obstetrics and gynecology of the Second Clinical Medical College of Army Medical University of China. Participants included a group of patients with low-risk CSP (10-point risk score <5, *n* = 23) and an equal-sized control group of patients having intrauterine pregnancy (IUP) with scar and electing to terminate pregnancy. Patients were excluded from the study if they had serious diseases of vital organs such as the heart, kidney, and lungs. Diagnosis of CSP was confirmed by review of sonographic images. In accordance with the “2013 revision of the Declaration of Helsinki,” all study participants gave written informed consent regarding study procedures and treatment modalities after the procedures had been fully explained to them.

**Figure 1:**
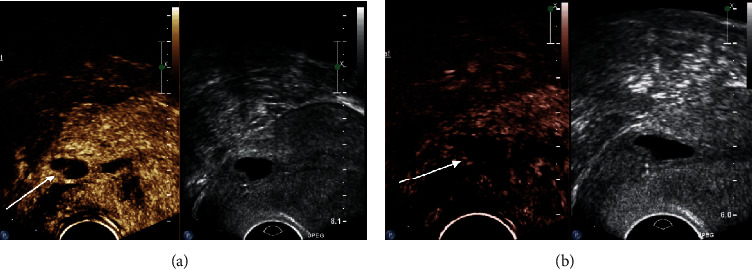
Findings from transvaginal contrast-enhanced ultrasonography before and after mifepristone administration in patients with low-risk cesarean scar pregnancy. (a) Before mifepristone treatment. (b) After mifepristone treatment.

**Figure 2:**
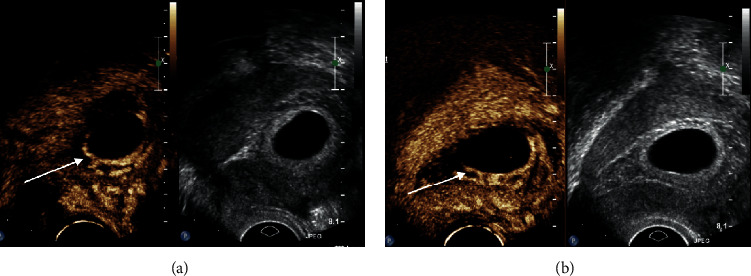
Findings from transvaginal contrast-enhanced ultrasonography before and after mifepristone administration in patients with intrauterine pregnancy. (a) Before mifepristone treatment. (b) After mifepristone treatment.

We assessed relevant demographic and clinical characteristics including age, parity, gestational age, BMI, and remnant myometrial thickness. All patients were prescribed 75 mg mifepristone daily for 2 days and underwent transvaginal CEUS before and after administration of mifepristone (Figures 1 and 2). On the third day, uterine curettage was performed after transvaginal CEUS.

### 2.1. CEUS Examination

All patients were examined by two obstetric ultrasound technicians with at least 5 years of experience. All ultrasound examinations were conducted using a Philips IU-22 system (Philips Electronics N.V., Amsterdam, Netherlands) with a 5–9 MHz transvaginal transducer. A 21G trocar was used to puncture the cubital vein and establish a venous channel. Next, 2.5 mL of the contrast agent was injected, and 5 mL of 0.9% normal saline was used for tube washing. When the contrast agent was injected, the patient began holding their breath and took a shallow breath when required or alternatively continued with slow shallow breathing. All patients were trained in the required breathing regime before the contrast process. Two minutes of ultrasound data were recorded and saved for analysis. The lesion area with the most evident enhancement was identified as the region of interest (ROI), with ROIs set as 5 mm diameter circles and remaining unchanged. The ROIs were located at the embryo decidua basalis. Related parameters obtained through the time intensity curve (TIC) included arrival time (AT, the time from injection of the agent to the point when the first contrast bubbles appeared in the gestational sac), peak intensity (PI, the maximal intensity of the TIC), and area under the curve around the gestational sac (AUC, the area under the TIC) [17].

Operations were ceased if vaginal bleeding exceeded 300 mL, in which case uterine balloons were employed for temporary hemostasis. It is recommended that UAE be performed if bleeding exceeds 500 mL. All study patients were followed up for at least one month following the study procedure; assessments included the serum *β*-hCG level and presence of abdominal pain, vaginal bleeding, and fever every month.

### 2.2. Evaluation of Curative Effects

Curative effects were assessed at three months following treatment and were rated as follows:

#### 2.2.1. Excellent Curative Effect

Ultrasound showed no residual gestational tissue; the patient had no abdominal pain, vaginal bleeding, or fever, and the serum *β*-hCG level decreased and returned to normal in three months.

#### 2.2.2. Moderate Curative Effect

Ultrasound results showed residual gestational tissue; the patient had abdominal pain, vaginal bleeding, or fever, and the serum *β*-hCG level was decreased. After recurettage or pharmaceutical treatment, ultrasound showed no residual gestational tissue, there were no symptoms such as abdominal pain, vaginal bleeding, or fever, and the serum *β*-hCG level returned to normal within three months.

#### 2.2.3. Poor Curative Effect

Ultrasound showed residual gestational tissue; the patient had abdominal pain, vaginal bleeding, or other symptoms, and the serum *β*-hCG level may have increased or decreased but did not decrease to normal levels. After recurettage or pharmaceutical treatment, ultrasound showed that the residual gestational tissue had persisted or grown. Patients had persistent vaginal bleeding or abdominal pain and needed further treatment such as laparoscopic surgery or UAE.

The total effective treatment rate was defined as the number of patients for whom treatment was rated as excellent or moderate divided by the total number of patients in each study group and expressed as a percentage.

### 2.3. Statistical Analysis

Analyses were performed using SPSS software version 16.0 (IBM, Armonk, NY, USA). All variables are presented as mean ± standard deviation. The paired *t*-test was used to compare patient characteristics between the CSP and IUP groups, to compare perfusion parameters as assessed by CEUS before vs. after mifepristone treatment, and to compare changes in perfusion parameters and curative effects between the two study groups. The chi-squared test was used to verify the efficacy of mifepristone. A *p* value of <0.05 was considered statistically significant.

## 3. Results

### 3.1. Patient Demographics and Clinical Characteristics

Patient characteristics for the CSP and IUP groups are shown in Table 1. Mean thickness of the lower uterine segment was significantly higher among women with IUP (5.72 ± 1.65 mm vs. 2.60 ± 1.20, *p* ≤ 0.001). There were no other significant differences between the two patient groups.

There were 16 patients with fetal heart activity in the CSP group and 15 patients in the IUP group. Two days after misoprostol administration, there were 9 patients without fetal heart activity detected in the CSP group and 7 patients without fetal heart activity in the IUP group (difference between the two groups not statistically significant, *χ*^2^ = 2.000; *p*=0.368).

Three patients in the CSP group reported pain without bleeding, 7 patients presented with bleeding but did not report pain, and 7 patients presented with both pain and bleeding. In the IUP group, 4 patients reported pain with no bleeding, 6 patients presented with bleeding but did not report pain, and 6 patients presented with both pain and bleeding.

### 3.2. CEUS Findings

CEUS perfusion quantification values for the two study groups before and after mifepristone treatment are shown in Table 2. In the CSP group, before mifepristone treatment, AT is 18.42 ± 3.38 (s), PI is 17.68 ± 2.84 (dB), and AUC is 1011.03 ± 194.53, and after mifepristone treatment, AT is 13.39 ± 1.98 (s), PI is 14.48 ± 2.81 (dB), and AUC is 800.33 ± 109.41. In IUP group, before mifepristone treatment, AT is 18.71 ± 2.01 (s), PI is 17.85 ± 2.61 (dB), and AUC is 1041.76 ± 168.14, and after mifepristone treatment, AT is 14.06 ± 2.85 (s), PI is 15.47 ± 2.44 (dB), and AUC is 878.49 ± 162.23. Based on TIC analysis, AT, PI, and AUC around the gestational sac were significantly lower in both study groups after mifepristone treatment than before (*p* < 0.05; Table 2).

Forty-eight hours following administration of mifepristone, in the CSP group, AT, PI, and AUC changes in blood flow around the gestational sac are 5.03 ± 2.97 (s), 0.01 ± 1.78 (dB), and 210.69 ± 121.14; in the IUP group, AT, PI, and AUC changes in blood flow around the gestational sac are 4.65 ± 3.09 (s), 0.15 ± 1.53 (dB), and 169.26 ± 74.06. Based on TIC analysis, changes in blood flow around the gestational sac, including AT, PI, and AUC, did not differ significantly between the two groups (Table 3).

### 3.3. Evaluation of Curative Effects

In the IUP group, no patients had vaginal bleeding >500 mL during uterine curettage. One week after uterine curettage, ultrasound showed no residual gestational tissue and no abdominal pain, vaginal bleeding, or fever, and serum *β*-hCG levels returned to normal in three months in all patients. Thus, the total effective treatment rate was 100% in the IUP group.

In the CSP group, two patients had vaginal bleeding >500 mL during uterine curettage; bleeding was significantly reduced following administration of 1 mL oxytocin during the operation, and no further treatment was needed. One week following the operation, 6 patients had residual gestational tissue, minimal vaginal bleeding, and no abdominal pain. These patients were instructed to take mifepristone orally. Among them, 2 patients underwent recurettage because there had been no evident reduction of gestational tissue. After recurettage, ultrasound showed no residual gestational tissue, there was no vaginal bleeding, and serum *β*-hCG levels returned to normal. One patient underwent laparoscopic resection after conservative treatment for 1 month because of persistent vaginal bleeding, continuous enlargement of residual gestational tissue, and disappearance of local muscular layer (Figure 2); there was no significant decrease in the serum *β*-hCG level in this patient. In the remaining 3 CSP patients with no abdominal pain, residual gestational tissue gradually shrank and returned to normal within one month, and serum *β*-hCG levels returned to normal within three months. The total effective treatment rate among CSP patients was thus 95.65% (Table 4). The effective treatment rate did not differ significantly between the two study groups (*χ*^2^ = 4.000; *p*=0.261).

## 4. Discussion

With continuing advances in research on CSP, it is now understood that the risk posed by CSP is affected by many factors, including the number of previous cesarean deliveries, the position of implantation of the gestational sac, and the timing within gestation [4, 5]. While numerous management options for CSP have been identified and evaluated, no standardized diagnostic or management guidelines have been developed [8, 9, 18]. Therefore, it is crucial to make an accurate diagnosis and to provide prompt therapy to avoid potentially catastrophic complications.

In our previous study evaluating the utility of a CSP risk scoring system to predict appropriate treatment, we found mifepristone combined with uterine curettage to be the optimal treatment for low-risk patients (those with a risk score <5) [10]. In the present study, we found no significant differences in maternal age, BMI, gravidity, parity, or gestational days between CSP and IUP patients, but average muscle layer thickness in the CSP group was less than half of that of the normal pregnancy group. Our finding of low remnant myometrial thickness in CSP is consistent with results from previous studies [9] and may stem from erosion of the muscular layer of the gestational sac when it is implanted in the scar, resulting in thinning of the gestational sac.

The effectiveness of high-dose mifepristone for abortion has been well-established [19–21]. Mifepristone influences the human endometrium during the luteal phase by reducing stromal edema, increasing venular diameter, and causing erythrocyte and leukocyte diapedesis and focal hemorrhage and degeneration of the stromal extracellular matrix. Through these mechanisms, eventual degradation of the endometrium is initiated, leading to termination of pregnancy.

CEUS has become a widely available and well-accepted imaging modality in recent years. By overcoming some of the limitations of conventional ultrasonography, CEUS creates a significant opportunity for visualization of the microcirculation [22]. Findings from the present study show that perfusion parameters around the gestational sac were significantly reduced following mifepristone treatment in both low-risk CSP patients and women with IUP. Accordingly, it appears that mifepristone brings about medical abortion in part through reducing microcirculation of the gestational sac by acting on endometrial vessels. In addition, changes in microcirculation of the gestational sac did not differ significantly between the two study groups, suggesting that mifepristone has the same effect on pregnancy termination in low-risk CSP as in normal pregnancy. Furthermore, both groups achieved similar curative effects through mifepristone combined with curettage. Accordingly, more aggressive treatments such as laparoscopy, hysteroscopy, and UAE can be avoided through the use of mifepristone combined with curettage in low-risk CSP patients. This conclusion is consistent with findings from Fu et al. [23] and suggests that personalizing treatment options based on the patient's condition can reduce the physical and mental impact of treatment on patients while also reducing the cost of their care.

Several limitations to our study should be acknowledged. First, this study was conducted at a single center, and our results should be confirmed in larger multicenter studies before being applied more widely in clinical practice. Second, we did not look at long‐term outcomes such as recurrent ectopic pregnancy or subsequent fertility. We recommend that future studies examine these outcomes in order to develop a richer understanding of the long-term safety and risks of mifepristone for treatment of low-risk CSP.

In conclusion, based on monitoring by CEUS, the effect of mifepristone in low-risk CSP was comparable to that in IUP, and combined with uterine curettage, we found this treatment was safe and effective in patients with low-risk CSP. In this patient population, such a treatment course can be used to avoid more aggressive treatments such as laparoscopy, hysteroscopy, and UAE.

## Figures and Tables

**Table 1:** Patient characteristics.

Characteristic	CSP (*n* = 23)	IUP (*n* = 23)	*t*	*p*
Maternal age (years)	32.26 ± 3.99	32.43 ± 3.78	0.152	0.880
BMI	23.12 ± 2.92	22.37 ± 3.96	−0.726	0.471
Gravidity	4.29 ± 1.43	4.61 ± 1.47	0.507	0.615
Parity	1.47 ± 0.51	1.52 ± 0.51	0.289	0.774
Diameter of gestational sac (mm)	20.40 ± 7.90	21.37 ± 11.64	0.331	0.742
Previous cesarean deliveries (times)	1.47 ± 0.51	1.52 ± 0.51	0.289	0.774
Thickness of the lower uterine segment (mm)	2.60 ± 1.20	5.72 ± 1.65	7.341	≤0.001

CSP, cesarean scar pregnancy; IUP, intrauterine pregnancy.

**Table 2:** CEUS perfusion quantification before and after mifepristone treatment in CSP and IUP patients.

Group	Parameter	Before mifepristone treatment	After mifepristone treatment	*t*	*p*
CSP (*n* = 23)	AT (s)	18.42 ± 3.38	13.39 ± 1.98	8.117	≤0.001
	PI (dB)	17.68 ± 2.84	14.48 ± 2.81	6.446	≤0.001
	AUC	1011.03 ± 194.53	800.33 ± 109.41	8.341	≤0.001
IUP (*n* = 23)	AT (s)	18.71 ± 2.01	14.06 ± 2.85	7.208	≤0.001
	PI (dB)	17.85 ± 2.61	15.47 ± 2.44	4.411	≤0.001
	AUC	1041.76 ± 168.14	878.49 ± 162.23	10.961	≤0.001

AT, arrival time; AUC, the area under the time intensity curve; CEUS, contrast-enhanced ultrasonography; CSP, cesarean scar pregnancy; IUP, intrauterine pregnancy; PI, peak intensity.

**Table 3:** Changes in blood flow around the gestational sac before vs. after mifepristone treatment in CSP and IUP patients.

Parameter	CSP (*n* = 23)	IUP (*n* = 23)	*t*	*p*
AT (s)	5.03 ± 2.97	4.65 ± 3.09	−0.424	0.674
PI (dB)	0.01 ± 1.78	0.15 ± 1.53	−1.115	0.271
AUC	210.69 ± 121.14	169.26 ± 74.06	−1.399	0.169

AT, arrival time; AUC, the area under the time intensity curve; CSP, cesarean scar pregnancy; IUP, intrauterine pregnancy; PI, peak intensity.

**Table 4:** Comparison of curative effects between CSP and IUP patients.

Group	*n*	Excellent curative effect	Moderate curative effect	Poor curative effect	Effective treatment rate (%)
CSP	23	16	6	1	95.65
IUP	23	23	0	0	100

CSP, cesarean scar pregnancy; IUP, intrauterine pregnancy.

## Data Availability

The data used to support the findings of this study are available from the corresponding author upon request.
